# Long-Term Advantages of Ovarian Reserve Maintenance and Follicle Development Using Adipose Tissue-Derived Stem Cells in Ovarian Tissue Transplantation

**DOI:** 10.3390/jcm9092980

**Published:** 2020-09-15

**Authors:** Luciana Cacciottola, Thu Y. T. Nguyen, Maria C. Chiti, Alessandra Camboni, Christiani A. Amorim, Jacques Donnez, Marie-Madeleine Dolmans

**Affiliations:** 1 Gynecology Research Unit, Institut de Recherche Expérimentale et Clinique, Université Catholique de Louvain, Av. Mounier 52, 1200 Brussels, Belgium; luciana.cacciottola@uclouvain.be (L.C.); thu.nguyen@uclouvain.be (T.Y.T.N.); maria.chiti@uclouvain.be (M.C.C.); alessandra.camboni@uclouvain.be (A.C.); christiani.amorim@uclouvain.be (C.A.A.); 2 Department of Anatomopathology, Cliniques Universitaires Saint-Luc, Av. Hippocrate 10, 1200 Brussels, Belgium; 3 Society for Research into Fertility, Av. Grandchamp 143, 1150 Brussels, Belgium; jacques.donnez@gmail.com; 4 Department of Gynecology, Cliniques Universitaires Saint-Luc, Av. Hippocrate 10, 1200 Brussels, Belgium

**Keywords:** ovarian tissue transplantation, adipose tissue-derived stem cells, ASCs, folliculogenesis, endocrine restoration, AMH, estradiol, long-term transplantation

## Abstract

(1) Background: Ovarian tissue transplantation with adipose tissue-derived stem cells (ASCs) has been shown to enhance graft vascularization and increase follicle survival after a short interval of 7 days. The aim of the present study was to investigate their long-term effects on primordial follicle pool maintenance and follicle development. (2) Methods: A total of 14 severe combined immunodeficient (SCID) mice were grafted with frozen-thawed human ovarian tissue with or without ASCs. Blood was taken monthly in order to quantify the anti-Müllerian hormone (AMH) and estradiol. After 6 months, all the grafts were retrieved and sent for histology and immunolabeling (AMH, AMH receptor II, estrogen receptors α and β, and c-kit/kit ligand). (3) Results: A significant upturn was observed in AMH and estradiol plasma levels 4 months after transplantation in both grafted groups. The primordial follicle pool was better preserved in the ASC group (41.86 ± 28.35) than in the standard transplantation group (9.65 ± 17.6, *p* < 0.05) compared to non-grafted controls (124.7 ± 140). (4) Conclusions: The use of ASCs prior to ovarian tissue transplantation yielded a larger primordial follicle pool and more physiological follicle distribution after long-term grafting. These findings suggested that ASC use might extend the ovarian tissue lifespan.

## 1. Introduction

Much scientific progress has been made in the treatment of malignancies in children and young adults over recent decades, yielding vastly improved (over 83%) 5-year overall survival rates in patients aged 0–19 years [1]. These positive data translate into a growing population of adult cancer survivors who may experience long-term consequences of chemotherapy and radiotherapy. Indeed, increased risks of premature ovarian failure and infertility in young women today require management by one of the currently available fertility restoration strategies [2]. Ovarian tissue cryopreservation (OTC) followed by transplantation (OTT) is an approach that has progressively gained ground, with more than 200 live births achieved so far, showing a logarithmic increase over the last two decades [3]. Moreover, it remains the only option for prepubertal girls and young women whose cancer treatment cannot be delayed [4,5].

Recently, a new two-step OTT technique has been developed using adipose tissue-derived stem cells (ASCs) to decrease early follicle loss due to hypoxia and ischemia after grafting [6]. The procedure boosts vascularization in the grafting site and leads to higher follicle survival rates 7 days post-transplantation [7,8]. The positive impact of ASCs in this specific model can be explained by their proangiogenic role, shortening the period of hypoxia [8], and improving revascularization by both vessel differentiation and adequate levels of vascular endothelial growth factor (VEGF) in ovarian grafts [9]. Follicle loss in transplanted ovarian tissue may also be due to massive follicle activation that occurs early after grafting and could be partially related to hypoxia-mediated cell signaling [10,11]. As follicle transition from primordial to primary is not reversible, this event strongly influences the primordial follicle pool and hence the graft lifespan. Activated follicle growth is then modulated by anti-Müllerian hormone (AMH), a member of the transforming growth factor-beta (TGF-β) superfamily produced by granulosa cells of growing follicles up to the antral stage, which acts as an inhibitor of granulosa cell proliferation and further follicle maturation and growth. The lack of secondary and tertiary follicles in grafted ovarian tissue and, consequently, AMH generation is responsible for accelerated follicle loss, also known as the burnout effect [12]. This event occurs in the first 3–6 months post-grafting [13] and results not only in accelerated follicle depletion but also disrupted synchrony between the oocyte and granulosa cells in follicle growth and differentiation [14].

Although the use of ASCs has been found to be effective in preserving the primordial follicle pool after short-term grafting, the impact of this transplantation strategy on long-term follicle growth and differentiation is still unknown. We aimed to investigate the mechanism by which preservation of the primordial follicle pool with ASCs may exert a long-term effect on follicle burnout and differentiation, as well as hormone generation and receptivity.

## 2. Experimental Section

### 2.1. Experimental Design

**Figure 1:**
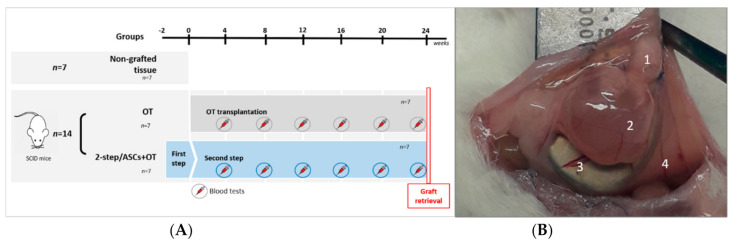
(**A**) Frozen-thawed ovarian tissue from 7 adult patients was distributed equally among the following groups: non-grafted controls, OT group (one-step transplantation to 7 SCID mice), and 2-step/ASCs+OT group (two-step transplantation to 7 SCID mice). The first step (ASCs loaded inside fibrin scaffolds) was carried out 2 weeks before the second step (OTT). Blood was taken every 4 weeks until euthanasia (after 24 weeks) and sample retrieval for histology and immunolabeling. (**B**) Human ovarian tissue retrieval from SCID mouse peritoneum after 6 months of transplantation in the 2-step/ASCs+OT group. Grafted ovarian tissue (1) with an antral follicle (2) and macroscopically visible vasculature (3) derived from mouse peritoneum (4). SCID: severe combined immunodeficient. ASCs: adipose tissue-derived stem cells. OT: ovarian tissue. OTT: ovarian tissue transplantation.

Fourteen severe combined immunodeficient (SCID) mice were used for this experiment (Figure 1A). They were ovariectomized and peritoneally xenografted with human ovarian tissue from 7 adult patients using either the two-step (2-step/ASCs+ovarian tissue [OT] group, *n* = 7) or one-step (OT group, *n* = 7) procedure, as reported by Manavella et al. [8]. In the 2-step/ASCs+OT group, a fibrin scaffold loaded with ASCs was first transplanted to prepare the grafting site two weeks prior to OTT. One fragment per patient was fixed after thawing to serve as controls for histological analysis (non-grafted controls group, *n* = 7).

Every month, blood was taken from each mouse for enzyme-linked immunosorbent assays (ELISA) in order to quantify AMH and estradiol generation in grafted ovarian tissue over time. After 24 weeks, all the ovarian fragments were retrieved for histology, immunohistochemistry (IHC), and immunofluorescence.

### 2.2. Adipose Tissue-Derived Stem Cells

Previously characterized human ASCs from female donors were commercially acquired (StemPRO human ASCs, Invitrogen, Carlsbad, CA, USA), cultured, and passaged, as described in an earlier publication [7], before being transplanted at high concentrations (1.5 × 10^6^) at passage 5.

### 2.3. Transplantation Procedure

Fourteen female SCID mice (Charles River Laboratories Écully, France) aged 9–11 weeks were used for this study. Animal welfare guidelines were followed, and the protocol was approved by the Committee on Animal Research of the Université Catholique de Louvain (2018/UCL/MD/40). Satisfactory housing and breeding conditions were maintained, as previously reported [15]. Anesthesia (ketamine 75 mg/kg; medetomidine 1 mg/kg) and analgesia (buprenorphine 0.1 mg/kg) were administered on every surgical procedure. Pain relief was also given after each blood test.

#### 2.3.1. Step 1: Preparation of the Peritoneal Grafting Site

Fibrin scaffolds were assembled, as previously described, with (i) 50 µL fibrinogen solution (50 mg/mL) (Tisseel, Baxter, Lessines, Belgium), (ii) 1.5 × 10^6^ ASCs suspended in 25 µL growth medium (Complete MesenPRO RSTM, Invitrogen, Carlsbad, CA, USA), and (iii) 50 µL thrombin (50 IU/mL) [16]. They were then incubated for 30–40 min. After making a median incision in the skin and peritoneum, fibrin implants were placed on scalpel-scratched peritoneal surfaces and sutured with two cardinal stitches using 6/0 Prolene, as detailed elsewhere [8].

#### 2.3.2. Human Ovarian Tissue Thawing Procedure

The Institutional Review Board of the Université Catholique de Louvain approved the use of human ovarian tissue for this study (2012/23Mar/125 num. B403201213872). Frozen ovarian tissue biopsies were taken from 7 women (age range 21–34 years) after obtaining their written informed consent. Ovarian tissue was frozen using the slow-freezing protocol, as already reported [17]. Patients had their ovarian tissue cryopreserved either for non-ovarian pathologies (1 cervical cancer, 1 breast cancer, 3 deep nodular endometriosis) or contralateral ovarian pathologies (2 monolateral borderline ovarian tumors). None of them had a history of ovarian surgery or gonadotoxic treatment before OTC. Cryogenic vials containing ovarian tissue fragments were thawed at room temperature for 2 min, immersed in a water bath at 37 °C for another 2 min, and then washed three times in fresh HEPES-MEM (Gibco, Borgloon, Belgium) to remove the cryoprotectant.

#### 2.3.3. Step 2: Ovarian Tissue Transplantation

Ovarian tissue biopsies from each patient were distributed equally between the different study groups: one fragment was fixed right after thawing (non-grafted control), one fragment was grafted following one-step OTT (OT group), and one fragment was used for the two-step procedure (2-step/ASCs+OT group). In the OT group, ovarian grafts were fixed to the peritoneum with 2 cardinal stitches, after scratching the peritoneal surface to improve graft attachment. In the 2-step/ASCs+OT group, the remaining fibrin implants were exposed, identified, and gently detached using blunt-tip forceps to create a peritoneal pocket into which ovarian tissue fragments were grafted [8].

### 2.4. ELISA

Blood was taken monthly from the tail tip of each mouse (60–90 μL). The plasma was isolated and kept at −20 °C until analysis. Human AMH and estradiol were measured in duplicate (using 10 μL of plasma for each measurement) every month from transplantation and bilateral ovariectomy to 24 weeks post-grafting using human AMH ELISA (Biomatik, Kitchener, ON, Canada) and estradiol ELISA (Invitrogen, Carlsbad, CA, USA) kits, according to the manufacturers’ instructions.

### 2.5. Histology and Immunolabeling

Ovarian samples from non-grafted controls and the 2-step/ASCs+OT and OT groups were fixed in 4% formaldehyde, embedded in paraffin, and serially sectioned (5 µm-thick sections). Every fifth slide from the first sample was stained with hematoxylin and eosin (H&E) (Merck, Darmstadt, Germany), and the rest were used for immunolabeling.

#### 2.5.1. Follicle Outcomes

H&E-stained slides were digitized using the Pannoramic P250 Flash III scanner, and the fragment surfaces were assessed by CaseViewer. Follicle density was determined by counting the number of follicles per mm^3^ on digitized slides in eleven random fragments per sample. Volume was calculated by multiplying digitized surfaces by fragment thickness. Only morphologically normal follicles with a visible oocyte (to avoid counting the same follicles twice) were taken into consideration [18]. Follicle survival was calculated as a percentage, comparing the follicle density of each graft with the follicle density of its corresponding non-grafted sample. Follicles were classified according to stage into primordial, primary, secondary, or antral [12], and the percentage of each stage was established for comparison between groups. Follicle atresia was evaluated using strict morphological criteria, namely ooplasm eosinophilia, granulosa cell pyknosis, cytoplasmic contraction, and/or the presence of vacuoles [18].

#### 2.5.2. Follicle Hormone Assessment

Follicle hormone activity was assessed using a number of markers: (i) estrogen receptors (ERs) α and β, both expressed in follicles from early stages, (ii) AMH, and (iii) AMH receptor II (AMHRII), the major effector of AMH signaling pathway activation.

Paraffin sections for each marker were deparaffinized with Histosafe (Yvsolab SA, Turnhout, Belgium) and rehydrated in 2-propanol (Merck, Darmstadt, Germany). After blocking endogenous peroxidase activity with 3% H_2_O_2_ and demasking in citrate buffer (pH 6) for 75 min at 98 °C, non-specific binding sites were blocked by incubation with normal goat serum for 30 min. Sections were first incubated overnight at 4 °C with primary antibodies, including rabbit polyclonal anti-ERα (1:400, MC-20 SC-542, Santa Cruz, CA, USA), rabbit polyclonal anti-ERβ (1:300, H-150 SC-8974, Santa Cruz, CA, USA), mouse anti-human AMH (1:100, MCA2246, Serotec, Gentaur, Kampenhout, Belgium), and rabbit polyclonal anti-AMHRII (1:200, 36093 Signalway Antibody, College Park, MD, USA). They were then incubated for one hour at room temperature with secondary antibodies, including EnVision rabbit HRP (1:1, K4003, Dako, USA) for ERα, ERβ, and AMHRII and EnVision mouse HRP (1:2, K4001, Dako, Carpinteria, CA, USA) for AMH. Diaminobenzidine (Dako, Carpinteria, CA, USA) was used as a chromogen. Negative controls consisted of the dilution solution without any primary antibodies (Supplementary Materials 1).

Stained slides were investigated for every marker at each follicle stage in all groups (non-grafted controls, 2-step/ASCs+OT, and OT), using the Leica SCN400 scanner at 20× magnification. Follicles at each stage were considered positive for markers when at least one granulosa cell was stained. Computer-assisted quantification of staining concentrations was then carried out for each marker with Leica’s proprietary artificial intelligence (Leica Biosystems, Wetzlar, Germany), as previously described [19].

#### 2.5.3. Follicle Competence and Maturation

The slides were subjected to double immunofluorescence targeting C-kit and kit ligand of human origin, in order to assess follicle maturation in terms of cross-talk between the oocyte and granulosa cells. Paraffin sections were deparaffinized with Histosafe (Yvsolab SA, Turnhout, Belgium) and rehydrated in 2-propanol (Merck, Darmstadt, Germany), before immersion in quenching buffer (NH4Cl, 50 nM). After blocking endogenous peroxidase activity with 3% H_2_O_2_ and demasking in Tris-EDTA (pH 9) for 20 min at 96 °C, non-specific binding sites were blocked by incubation with normal goat serum for 30 min. Sections were incubated at overnight 4 °C with the first primary antibody, polyclonal rabbit anti-human c-kit (1:200, A4502, lot 10115388, Dako, Carpinteria, CA, USA), and for one hour at room temperature with the secondary antibody, EnVision rabbit HRP (1:1, K4003, Dako, Carpinteria, CA, USA). They were then incubated for 15 min at room temperature in boric acid solution with 1:1000 H_2_O_2_ at 30% and 1:200 Alexa Fluor 488 tyramide reagent (B40953, lot 2051204, Invitrogen, Carlsbad, CA, USA) as the chromogen, followed by a 20-min citrate buffer bath in the microwave.

After once again blocking nonspecific binding sites with normal goat serum for 30 min, the slides were incubated overnight at 4 °C with the second primary antibody, mouse anti-human Kit ligand (1:25, clone G-3, SC-13126, Santa Cruz, CA, USA), and for one hour at room temperature with the secondary antibody, EnVision mouse HRP (1:1, K4001, Dako, Carpinteria, CA, USA). They were then incubated for 15 min at room temperature in boric acid solution with 1:1000 H_2_O_2_ at 30% and 1:200 Alexa Fluor 647 tyramide reagent (B40958, lot 2009264, Invitrogen, Carlsbad, CA, USA) as the chromogen. Final incubation in Hoechst 333452 (1:1000, H3570, lot 1840439, Invitrogen, Carlsbad, CA, USA) ensured staining of cell nuclei. The slides were mounted using the HIGHDEF IHC fluoromount (Enzo, New York, NY, USA). Negative controls were incubated with secondary antibodies only.

The sections were then digitized with Pannoramic P250 Flash III using the following filters: green (SR-FITC-Zero) for Alexa Fluor 488, orange (SP-CY5–4040c-Zero) for Alexa Fluor 647, and blue (DAPI-5060c-000-Zero) for Hoechst 33342. Follicles at each stage were considered positive for c-kit when the oocyte cytoplasm was stained and for kit ligand when at least one granulosa cell was positive. Antral follicle analysis was not possible as the majority of selected sections did not show an oocyte inside follicles.

### 2.6. Statistical Analysis

Results were presented as means ± SEM. GraphPad Prism, version 8 for Windows (GraphPad Software, San Diego, CA, USA) was used for statistical analysis. Two-way analysis of variance (ANOVA), one-way analysis of variance (ANOVA), or Kruskal–Wallis tests were conducted depending on normality distribution criteria. Tukey’s or Fisher’s post hoc Least Significant Difference (LSD) multiple comparisons were applied where appropriate. The *p*-values <0.05 were considered statistically significant.

## 3. Results

### 3.1. Graft Recovery Rate and Macroscopic Aspect

All grafted tissues (100%) were retrieved from the 14 mice. Three grafts in the 2-step/ASCs+OT group and one in the OT group contained macroscopically visible antral follicles larger than 5 mm in size. Some blood vessels were observed connecting ovarian fragments with peritoneum (Figure 1B).

### 3.2. Hormone Kinetics

**Figure 2:**
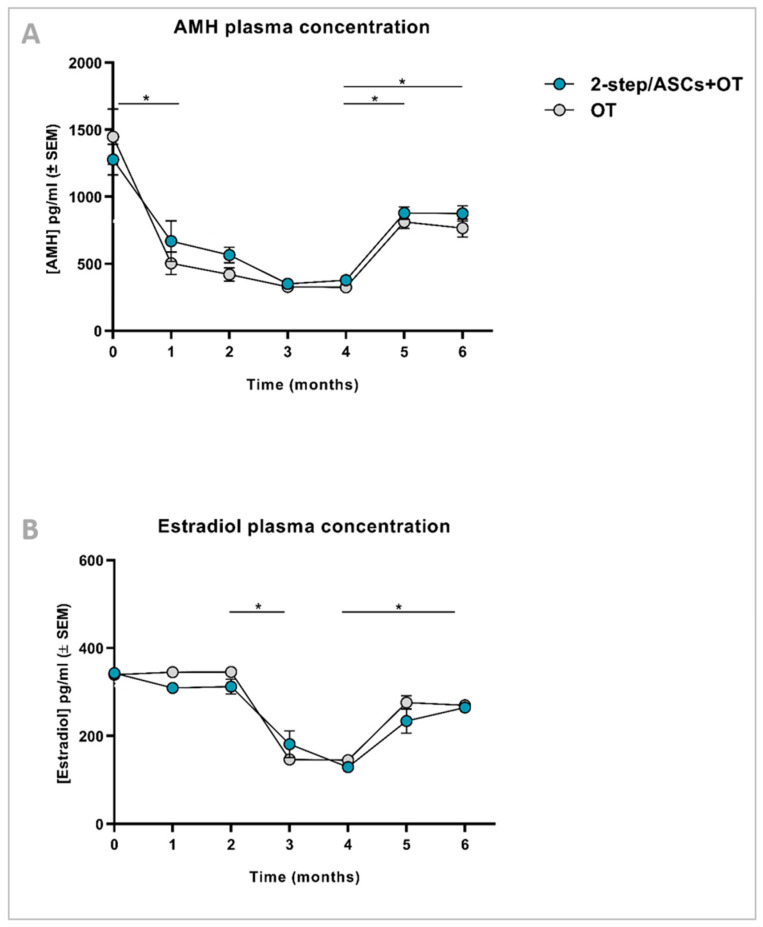
AMH (**A**) and estradiol (**B**) concentrations (pg/mL ± SEM) in mouse plasma ELISA assays were analyzed every 4 weeks by one-way analysis of variance (ANOVA) and Tukey’s post hoc test. Significant differences between time points are indicated as follows: * *p* < 0.05. No differences were detected between groups (OT vs. 2-step/ASCs+OT). AMH: anti-Müllerian hormone. ELISA: enzyme-linked immunosorbent assay. OT: ovarian tissue. ASCs: adipose tissue-derived stem cells.

AMH and estradiol concentrations were quantified in murine plasma every month, and the kinetic curves were constructed for each (Figure 2). High levels of detection of both hormones during surgery for xenotransplantation and ovariectomy (time point (TP) 0) were due to murine AMH and estradiol cross-reactivity, as demonstrated in other studies [20]. AMH values decreased after one month, as murine AMH was progressively washed out, then remained detectable at low levels until month 4, before increasing again 5 months post-transplantation (TP4 vs. TP5: *p* < 0.05; TP4 vs. TP6 *p* < 0.05). No difference was found between 2-step/ASCs+OT and OT groups at any TP (Figure 2A). Estradiol levels remained stable longer than AMH, then fell significantly from month 2 to month 3 (TP2 vs. TP3: *p* < 0.05) and rose again at month 5 (TP4 vs. TP5: *p* < 0.05). No difference was observed between the 2-step/ASCs+OT and OT groups at any TP (Figure 2B).

### 3.3. Follicle Outcomes

**Figure 3:**
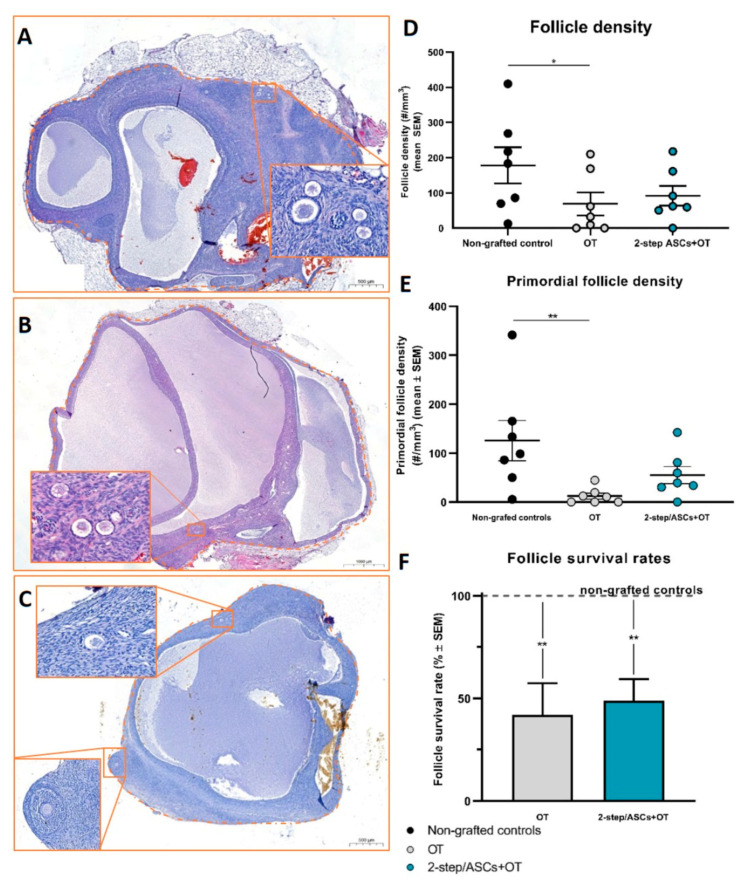
H&E sections of 6-month grafted tissues showed antral and primordial follicles in the 2-step/ASCs+OT group (**A**,**B**) and antral and secondary follicles in the OT group (**C**). Scale bars: 1000 µm and 20 µm. (**D**) Follicle density (mean number of follicles/mm^3^) and (**E**) primordial follicle density (mean number of follicles/mm^3^) were compared between groups (non-grafted controls, OT, and 2-step/ASCs+OT) using two-way ANOVA and Tukey’s post hoc test. (**F**) Follicle survival rates (percentage, mean ± SEM) were compared between groups (non-grafted controls, OT, and 2-step/ASCs+OT) using one-way ANOVA and Tukey’s post hoc test. Significant differences between groups are indicated as follows: * *p* < 0.05; ** *p* < 0.01. H&E: hematoxylin and eosin. OT: ovarian tissue. ASCs: adipose tissue-derived stem cells.

A total of 436 follicles were counted in H&E-stained sections (Figure 3A–C): (i) 210 in non-grafted controls; (ii) 169 in the 2-step/ASCs+OT group; (iii) 57 in the OT group (Table S1). Follicle density was evaluated as follicles/mm^3^ (mean ± SEM). Follicle density was significantly lower in the OT group (69 ± 32.53) than in non-grafted controls (178.3 ± 51.42, *p* = 0.02), while no significant difference was observed between the 2-step/ASCs+OT group (91.85 ± 27.94) and non-grafted controls (Figure 3D). Additional analysis was conducted to assess differences in follicle survival rates, determined as a percentage, and compare non-grafted control tissues (considered as 100%) with those in each corresponding grafted fragment. Follicle survival rates (mean% ± SEM) were significantly lower in both grafted groups than in non-grafted controls (OT: 42.01 ± 15.34, *p* = 0.003; 2-step/ASCs+OT: 48.89 ± 10.58, *p* = 0.009) (Figure 3F). Primordial follicle density (primordial follicles/mm^3^, mean ± SEM) was also significantly lower in the OT group (12.46 ± 6.06) than in non-grafted controls (125.8 ± 41.02, *p* = 0.002), but no significant difference was found between the 2-step/ASCs+OT procedure (55.1 ± 17.34) and non-grafted controls (Figure 3E).

**Figure 4:**
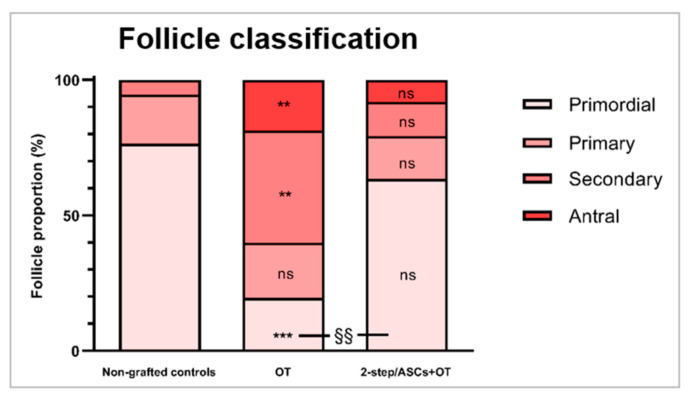
Stacked percentages of each follicle stage (primordial, primary, secondary, and antral) were compared between groups (non-grafted controls, OT, and 2-step/ASCs+OT) with the one-way ANOVA and Tukey’s post hoc test. Differences between the grafted groups and non-grafted controls: ** *p* < 0.01, *** *p* < 0.0001. Differences between the OT and 2-step/ASC+OT groups: §§ *p* < 0.01). OT: ovarian tissue. ASCs: adipose tissue-derived stem cells. ns: not significant.

In terms of follicle classification and comparison of follicle stages, the percentage of primordial follicles (mean ± SEM) was significantly lower in the OT group (19.48 ± 6.85%) compared to non-grafted controls (76.46 ± 6.87%, *p* = 0.0003) and the 2-step/ASCs+OT group (63.48 ± 9.04%, *p* = 0.004). Indeed, similar primordial follicle proportions were detected between the 2-step/ASCs+OT group and non-grafted controls. The percentage of primary follicles did not differ significantly between groups (non-grafted controls: 18.11 ± 4.14%; 2-step/ASCs+OT: 15.91 ± 5.24%; OT: 20.48 ± 8.41%). On the contrary, the percentage of secondary follicles was significantly higher in the OT group (41.38 ± 16.43%) than in non-grafted controls (5.43 ± 3.42%, *p* = 0.003), while no significant difference was detected in the 2-step/ASCs+OT group (12.48 ± 5.03%). Antral follicles were only encountered in grafted tissues: 22 antral follicles in the 2-step/ASCs+OT group (8.13 ± 3.11%), and 11 antral follicles in the OT group (18.6 ± 7.05, *p* = 0.009) (Figure 4).

### 3.4. AMH Expression

#### 3.4.1. Positive Follicle Count

A total of 170 follicles were analyzed: 77 from non-grafted controls, 52 from the 2-step/ASCs+OT group, and 41 from the OT group. Positive immunostaining for AMH (at least one positive granulosa cell per follicle) was observed at all follicle stages (Table 1). Similar percentages of AMH-positive primordial and secondary follicles were found in both grafted groups compared to non-grafted controls. Statistically higher AMH-positive percentages were detected in primary follicles in the OT group compared to non-grafted controls (100% vs. 32.5% *p* = 0.01).

#### 3.4.2. Quantification of Staining Concentrations

**Figure 5:**
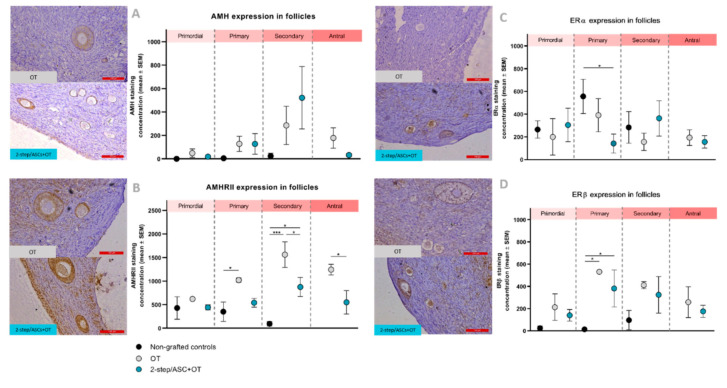
(**A**) AMH, (**B**) AMHRII, (**C**) ERα, and (**D**) ERβ staining concentrations (mean ± SEM) comparisons between groups (non-grafted controls, OT, and 2-step/ASCs+OT), and follicle stages (primordial, primary, secondary, and antral) were analyzed using Kruskal–Wallis and Fisher’s post hoc LSD tests. Significant differences between groups are indicated as follows: * *p* < 0.05; *** *p* < 0.001. AMH: anti-Müllerian hormone. AMHRII: anti-Müllerian hormone receptor type II. ER: estrogen receptor. OT: ovarian tissue. ASCs: adipose tissue-derived stem cells. Scale bars: 100 µm.

Similar AMH staining concentrations were observed in primordial follicles across all groups. AMH levels showed an upward trend in primary and secondary follicles of grafted tissues compared to non-grafted controls, but no statistical difference was encountered between groups (Figure 5A).

### 3.5. AMHRII Expression

#### 3.5.1. Positive Follicle Count

A total of 137 follicles were analyzed: 51 from non-grafted controls, 53 from the 2-step/ASCs+OT group, and 33 from the OT group. Positive immunostaining for AMHRII (at least one positive granulosa cell per follicle) was observed at all follicle stages (Table 1). No significant differences were noted in AMHRII-positive follicle percentages between any of the groups.

#### 3.5.2. Quantification of Staining Concentrations

Similar AMHRII staining concentrations were found in primordial follicles across all groups. Significantly greater expression was identified in primary follicles in the OT group compared to non-grafted controls (*p* = 0.04), but no difference was detected between the latter and the 2-step/ASCs+OT group. Both grafted groups showed significantly increased AMHRII expression in secondary follicles compared to non-grafted controls (2-step/ASCs+OT: *p* = 0.02, OT: *p* = 0.0002), and higher AMHRII levels were observed in the OT group than the 2-step/ASCs+OT group (*p* = 0.02). In antral follicles too, significantly elevated AMHRII expression was found in the OT group compared to the 2-step/ASCs+OT group (*p* = 0.01) (Figure 5B).

### 3.6. ERα Expression

#### 3.6.1. Positive Follicle Count

A total of 181 follicles were analyzed: 87 from non-grafted controls, 47 from the 2-step/ASCs+OT group, and 47 from the OT group. Positive immunostaining for ERα (at least one positive granulosa cell per follicle) was observed at all follicle stages (Table 1). Fewer ERα-positive primary follicles were detected in the two grafted tissue groups than in non-grafted controls, with a statistically significant decrease in the 2-step/ASCs+OT group (non-grafted controls vs. 2-step/ASCs+OT: 100% vs. 47%, *p* = 0.03). Fewer ERα-positive secondary follicles were encountered in the grafted tissue groups compared to non-grafted controls, with a statistically significant downturn in the OT group (non-grafted controls vs. OT: 100% vs. 55%, *p* = 0.03).

#### 3.6.2. Quantification of Staining Concentrations

Similar ERα staining concentrations were observed in primordial follicles across all groups. Lower ERα staining levels were found in primary follicles of both grafted groups, but this was only statistically significant when comparing the 2-step/ASCs+OT group and non-grafted controls (*p* = 0.01) (Figure 5C). ERα staining concentrations were also similar in secondary and antral follicles across all groups.

### 3.7. ERβ Expression

#### 3.7.1. Positive Follicle Count

A total of 156 follicles were analyzed: 63 from non-grafted controls, 54 from the 2-step/ASCs+OT group, and 39 from the OT group. Positive immunostaining for ERβ (at least one positive granulosa cell per follicle) was observed at all follicle stages (Table 1). Significantly more ERβ-positive primordial follicles were detected in both grafted tissue groups (non-grafted controls vs. 2-step/ASCs+OT: 31% vs. 80%, *p* = 0.04; non-grafted controls vs. OT: 31% vs. 100%, *p* = 0.002) than in non-grafted controls. Higher ERβ-positive levels were also identified in primary follicles of grafted tissues, reaching statistical significance in the 2-step/ASCs+OT group compared to non-grafted controls (non-grafted controls vs. 2-step/ASCs+OT: 54% vs. 100%, *p* = 0.03).

#### 3.7.2. Quantification of Staining Concentrations

An upward trend in ERβ staining concentrations was detected at all follicle stages after grafting. Significantly higher ERβ staining levels were observed in primary follicles of both grafted groups compared to non-grafted controls (Figure 5D). No significant difference was found between the 2-step/ASCs+OT and the OT groups.

### 3.8. C-Kit and Kit Ligand Expression

Qualitative evaluation of c-kit and kit ligand expression was conducted in primordial, primary, and secondary follicles. Follicles at each stage were considered positive for c-kit when the oocyte cytoplasm showed staining and for kit ligand when at least one granulosa cell was positive. A total of 77 follicles were analyzed: 67 from non-grafted controls, 60 from the 2-step/ASCs+OT group, and 58 from the OT group (Table 1).

Positive staining for c-kit was detected at all follicle stages in each group. An upward trend was identified in grafted tissues at all follicle stages compared to non-grafted controls. In the OT group, c-kit staining was significantly greater than in non-grafted controls. Positive staining for kit ligand was only found in primary and secondary follicles of grafted tissues. No primordial follicles were positive for kit ligand in any of the groups. An upward trend was observed in the 2-step/ASCs+OT group compared to non-grafted controls and the OT group, but it was not statistically significant.

## 4. Discussion

In this paper, we demonstrated that the 2-step transplantation technique using ASCs results in better primordial follicle preservation. Looking at the literature, a number of strategies have been implemented in an attempt to improve follicle outcomes after OTT by acting on the neovascularization of ovarian grafts. Among them, administration of growth factors, such as VEGF [21], hormones, such as erythropoietin [22], and angiogenesis modulators, such as sphingosine-1-phosphate [23], have shown partial beneficial effects on follicle survival.

Over the years, the idea of taking advantage of granulation tissue by transplanting the ovarian cortex in two steps to enhance neovascularization has begun to take shape and yielded promising results [24,25]. Following this lead, we set out to prepare the transplantation site for ovarian tissue grafts using ASCs, which proved to be an effective strategy for reducing initial hypoxia and ischemia damage shortly after transplantation. In a first study, we demonstrated that ASCs, at specific concentrations, were able to differentiate into endothelial lineages when transplanted to the peritoneum [7]. We established, in a second study, their effectiveness at shortening hypoxia, mitigating follicle loss, and promoting follicle survival 7 days post-grafting [8]. In a third study, we investigated the mechanisms by which ASCs exert their effects on transplanted tissue to improve outcomes. We thus confirmed their ability to induce angiogenesis within a short interval by differentiating into endothelial-like cells and boosting proangiogenic growth factors in revascularized areas [9,26,27]. Having developed such a promising strategy to enhance OTT results [28], the aim of the present study was to investigate the long-term impact of ASCs on ovarian tissue viability and lifespan in terms of follicle survival, development, and endocrine resumption.

### 4.1. Follicle Density and Primordial Follicle Survival

Endocrine resumption is observed in more than 90% of women 3–6 months after OTT, with some variation in the duration of endocrine activity and subsequent follicle growth, depending on the ovarian reserve [29,30,31]. In our group, the mean duration of transplanted tissue function is 5–6 years [4,29], contingent on a number of factors: (i) the age of patients at the time of tissue cryopreservation and their baseline ovarian reserve; (ii) the quality of freezing, thawing, and transplantation procedures; (iii) the amount of grafted tissue present; (iv) the degree of ischemia and hypoxia post-transplantation [4,32].

Ovarian function and longevity after grafting are mainly dependent on the follicle pool, especially primordial follicles, which, over time, are able to cede their quiescent status and develop enough to reach ovulation [33]. It is, therefore, vital to preserving the ovarian reserve at each clinical step, from surgical ovarian tissue retrieval to grafting of ovarian fragments. According to previous studies, two main events are responsible for the loss of around 50–90% of follicles after transplantation [15,34], namely follicle death due to hypoxia/ischemia [6] and massive follicle activation [11,35]. Indeed, not only do we witness a significant decrease in follicle density after human OTT due to hypoxia but also large-scale primordial follicle activation, with an increase in growing follicles as early as 3 days after grafting through the phosphatidylinositol 3-kinase (PI3K)/protein kinase B (Akt) pathway [11,35].

Our team already proved that use of ASCs prior to transplantation of ovarian fragments was able to shorten the hypoxic period and boost revascularization [8,9]. When scrutinizing follicle outcomes in these studies, we could observe significantly higher follicle density and also lower proportions of both atretic and growing follicles in the ASC group 7 days after OTT [8]. These initial findings suggested a role for ASCs in maintaining the quiescent state of primordial follicles through their effect on hypoxia/ischemia. In the present study, similar follicle survival rates were obtained after long-term transplantation between grafted groups, but when considering follicle stages, the primordial follicle pool appeared to be better maintained over time in the ASC group. In terms of follicle distribution, we found 20% primordial follicles, 20% primary follicles, 42% secondary follicles, and 18% antral follicles in the OT group, so after the standard OTT, the primordial follicle pool was less represented in our findings than in other published studies. However, this value is extremely variable, ranging from 36% [13] to 92% [36], depending on the absolute values of the follicle reserve. When considering follicle distribution in our specific patient group, who did not have a rich follicle reserve, similar values were observed in the 2-step/ASCs+OT group and in non-grafted controls in both quiescent and growing follicles. These findings suggested physiological follicle distribution with the use of ASCs after long-term xenografting.

On the contrary, in standard OTT, the primordial follicle pool was almost lost after 6 months, with significantly lower follicle density and significantly higher proportions of growing follicles than in non-grafted controls. Our recent results were able to confirm the ability of ASCs to preserve the primordial follicle pool after long-term transplantation too, so the impact of ASCs in protecting primordial follicles from both death and abnormal activation soon after transplantation appears to be maintained over time. These beneficial effects may yield a longer graft lifespan, thanks to the more efficient preservation of the primordial follicle reserve.

### 4.2. AMH and AMHRII

In our experimental design, AMH was chosen as a marker of follicle growth in grafted human ovarian tissue, as it is produced by granulosa cells of developing follicles up to the antral stage. Its receptor AMHRII was also included in our analysis since it is the major effector of AMH signaling pathway activation. The AMH-mediated signaling pathway plays a known role in follicle growth and maintenance of homeostasis of the preantral follicle reserve. It is also responsible for follicle sensitivity to gonadotropins, inhibin B production, and steroidogenic activity modulation [37]. AMH exerts its effect through AMHRII, mainly responsible for the phosphorylation of signaling pathway effectors of the Smad family [38]. AMHRII expression appears to decrease when AMH levels increase, suggesting negative feedback, as demonstrated in vivo using recombinant AMH [20].

Our results showed AMH and AMHRII expression at all follicle stages, irrespective of transplantation. Previous studies have already described the presence of AMH in primordial follicles of both primates and humans [13,39,40]. AMHRII gene expression has also been reported at all follicle stages, including primordial, in humans [39], but data on its detection by IHC were lacking. Despite the presence of the AMH signaling pathway in primordial follicles, our findings did not suggest any effect of this hormone on their maintenance.

Plasma AMH kinetics were similar over time in both grafted groups, as confirmed by comparable absolute numbers of secondary and antral follicles (Supplementary Table 1). As different primordial follicle pools were observed in our study between the grafted groups, with such analogous AMH kinetics, we may conclude that AMH does not appear to play a role in protecting primordial follicles from accelerated growth. These findings confirmed clinical observations that AMH is not a good marker of graft function duration or fertility potential for transplanted patients [41].

Regarding AMHRII expression, greater staining intensity was observed in growing follicles in the standard transplantation group than in the ASC group. As these results were detected in the presence of similar AMH levels, we can hypothesize that feedback between AMH and its receptor may be regulated by other factors. These could increase AMHRII expression in case of an imbalance between quiescent and growing follicles in order to slow the pace of follicle burnout. Nevertheless, further studies should be conducted to investigate AMH-mediated signaling during follicle development.

### 4.3. Estradiol and ERs

Estradiol generation is a key activity in follicles, as it results in positive modulation of follicle growth at all stages, and also gonadal axis stimulation, with consequent menstrual cycle resumption in transplanted patients. In vitro studies on isolated follicles suggest that steroidogenic activity may be disrupted by massive follicle activation, leading to impaired follicle quality [42], but there are no in vivo studies to confirm these findings. To investigate estradiol-mediated signaling in grafted human ovarian tissue, we quantified estradiol generation in grafts over time, as well as its receptor expression at all follicle stages 6 months post-transplantation. Both receptor isoforms, ERα and ERβ, were investigated. These proteins are highly homologous with different distributions according to the tissue [43]. In the ovary, ERα is the major effector of estrogen action on early follicle growth [44]. ERβ, on the other hand, serves as an effector of estrogen-dependent differentiation and later-stage growth [45].

Our findings detected no difference in estradiol kinetics over time between the two grafted groups. Estradiol values did not reflect follicle proportions in grafted tissues as more growing follicles were observed after standard OTT, with similar values between groups. Moreover, follicles already at the growth stage at the time of transplantation were less able to survive because of their higher metabolic demands, which could not meet before the tissue made a full recovery from hypoxic damage. These follicles were probably lost after grafting, while the second wave of follicle recruitment is thought to progressively lead to antral follicle development after 6 months [13]. This hypothesis may explain the delayed drop in estradiol production in the first 2 months post-transplantation, which could be caused by granulosa cell activity from the first growth wave of follicles, while the late upturn is likely due to physiological antral follicle development in human ovarian tissue [13].

Regarding ERα, which is responsible for the early growth and proliferation of granulosa cells, similar expression was observed in primordial follicles of both grafted groups compared to non-grafted controls. Primordial follicle responsiveness to estradiol-mediated signaling, therefore, appeared to be preserved after long-term grafting, favoring future growth, irrespective of transplantation technique. Moreover, an inverse trend was noted between ER isoforms in growing follicles in both grafted groups compared to non-grafted controls, with a decrease in ERα and an increase in ERβ. As ERβ-mediated signaling plays a major role in follicle maturation, its increased expression may be a sign of ongoing follicle development after long-term transplantation.

### 4.4. C-Kit and Kit Ligand

C-kit/kit ligand signaling is expressed in human growing follicles and is crucial to follicle development and oocyte maturation [46,47]. The kit ligand binds to its receptor c-kit to activate different signaling pathways, like the PI3K pathway for regulation of cell survival and proliferation [48]. Higher c-kit expression in primordial follicles may indicate loss of quiescence after standard transplantation, which is consistent with follicle stage proportions in favor of growth stages in this group. We hoped that the c-kit/kit ligand signaling pathway could be used as a marker of oocyte quality, but no significant difference was found in kit ligand expression between grafted tissues and non-grafted controls.

### 4.5. Limitations of The Study

We acknowledge that there are some limitations to our study. The first is due to the high intra- and inter-sample variability in follicle distribution in human ovarian tissue. For this reason, even if we provided enough data to support our hypothesis of an impact of ASCs on the primordial follicle reserve, it would still not deliver clear answers about all aspects of ovarian tissue resumption and behavior after transplantation. Further studies will, therefore, be needed to broaden our knowledge of endocrine resumption mechanisms in native and transplantation-related folliculogenesis. In this study, however, the application of power analysis for sample size determination may have resulted in excessive resource use, with no counterbalanced benefits. The second obstacle for all research groups in this field is related to the limited availability of human ovarian tissue from patients of reproductive age, with the obvious need to keep its use to a minimum.

## 5. Conclusions

We demonstrated, for the first time, that two-step transplantation of ovarian tissue with ASCs resulted in better long-term primordial follicle preservation, although ASCs did not appear to directly modulate hormone production for endocrine resumption or antral follicle development. The primordial follicle pool remained unaffected by AMH- and estradiol-mediated pathways, which did not change irrespective of the transplantation technique used. Indeed, its maintenance looks to be dependent on the degree of hypoxia/ischemia after grafting, which can be reduced by adequate preparation of the transplantation site with ASCs.

## Figures and Tables

**Table 1:** For each immunolabeling (AMH, AMHRII, ERα, ERβ, c-kit, and kit ligand), values show the mean ± SEM for percentages of positive-stained follicles (parentheses show the number of positive follicles out of the total number of follicles counted), analyzed using the Kruskal–Wallis and Fisher’s post hoc LSD tests.

	Primordial			Primary			Secondary			Antral		
	Non-grafted controls (%)	OT (%)	2-step/ ASCs+OT (%)	Non-grafted controls (%)	OT (%)	2-step/ ASCs+OT (%)	Non-grafted controls (%)	OT (%)	2-step/ ASCs+OT (%)	Non-grafted controls (%)	OT (%)	2-step/ ASCs+OT (%)
AMH	6.2 ± 6.2	44 ± 29.4	50 ± 28.8	32.5 ± 13.1 a	100 ± 0 b	80.6 ± 15.5	33 ± 17	94 ± 6	88.6 ± 11.3	0	98 ± 2	90.3 ± 9.6
	(3/40)	(2/5)	(4/8)	(7/29)	(8/8)	(12/17)	(2/8)	(11/12)	(12/14)		(15/16)	(11/13)
AMHRII	80 ± 20	100 ± 0	100 ± 0	90 ± 10	100 ± 0	100 ± 0	100 ± 0	100 ± 0	100 ± 0	0	100 ± 0	100 ± 0
	(13/29)	(1/1)	(7/7)	(12/16)	(4/4)	(9/9)	(6/6)	(13/13)	(23/23)		(15/15)	(14/14)
ERα	76 ± 24	70 ± 30	83.2 ± 16.7	100 ± 0 a	75 ± 25	47.7 ± 22.1 b	100 ± 0 a	55.3 ± 5.3	75 ± 25 b	0	85.2 ± 14.7	90 ± 10
	(50/55)	(3/6)	(6/10)	(19/19)	(7/11)	(5/10)	(13/13)	(9/14)	(10/16)		(9/16)	(9/11)
ERβ	31.7 ± 11.8 a	100 ± 0 b	80 ± 20 b	54 ± 29.1 a	100 ± 0	100 ± 0 b	75 ± 25	93.3 ± 6.6	100 ± 0	0	97.5 ± 2.5	100 ± 0
	(16/49)	(5/5)	(10/13)	(6/10)	(6/6)	(8/8)	(3/4)	(13/14)	(18/18)		(13/14)	(15/15)
C-kit	22.1 ± 89.7 a	100 ± 0 b	57.9 ± 19.7	37.2 ± 13.5	90 ± 10	75.7 ± 12.6	23.8 ± 23.8	68.5 ± 10.8	57.7 ± 20.1	N/A	N/A	N/A
	(11/33)	(7/7)	(16/21)	(10/22)	(12/15)	(13/17)	(5/12)	(15/36)	(15/22)			
Kit ligand	0	0	0	3.1 ± 3.1	0	27 ± 10.4	4.7 ± 4.7	19.7 ± 6.6	36 ± 13.7	N/A	N/A	N/A
	(0/33)	(0/7)	(0/21)	(1/22)	(0/15)	(2/17)	(1/12)	(6/36)	(9/22)			

N/A: no analysis was conducted. AMH: anti-Müllerian hormone. AMHRII: anti-Müllerian hormone receptor type II. ER: estrogen receptor. Statistical differences are indicated by different letters (a≠b = *p* < 0.05).

## Supplementary Materials

The following are available online at https://www.mdpi.com/2077-0383/9/9/2980/s1, Supplementary Materials 1: Immunostaining protocols, Table S1: Total and per patient follicle count in non-grafted controls, the 2-step/ASCs+OT group, and the OT group.
