# Supplementary material for: Long-Term Advantages of Ovarian Reserve Maintenance and Follicle Development Using Adipose Tissue-Derived Stem Cells in Ovarian Tissue Transplantation
Source: J Clin Med. 2020 Sep 15;9(9):2980. doi: 10.3390/jcm9092980 (PMC7564479; doi:10.3390/jcm9092980)
Supplement: Supplementary file 1 [file jcm-09-02980-s001.pdf]

Supplementary materials 1. Immunostaining protocols

ERα immunostaining protocol

Sections were deparaffinized with Histosafe (Yvsolab SA, Turnhout, Belgium) and rehydrated in 2-propanol (Merck, Darmstadt, Germany). After blocking endogenous peroxidase activity with 3% H_2O_2 and demasking in citrate buffer (pH 6) for 75 min at 98 °C, nonspecific binding sites were blocked by incubation with normal goat serum for 30 min. Sections were first incubated overnight at 4 °C with rabbit polyclonal anti-ERα antibody (1:400, MC-20 SC-542, Santa Cruz, CA, USA) and then for 1 h at room temperature with Envision rabbit HRP (1:1, K4003, Dako, Carpinteria, CA, USA). Diaminobenzidine (Dako, Carpinteria, CA, USA) was used as a chromogen and hematoxylin as a counterstain.

ERβ immunostaining protocol

Sections were deparaffinized with Histosafe (Yvsolab SA, Turnhout, Belgium) and rehydrated in 2-propanol (Merck, Darmstadt, Germany). After blocking endogenous peroxidase activity with 3% H_2O_2 and demasking in citrate buffer (pH 6) for 75 min at 98 °C, nonspecific binding sites were blocked by incubation with normal goat serum for 30 min. Sections were first incubated overnight at 4 °C with rabbit polyclonal anti-ERβ antibody (1:300, H-150 SC-8974, Santa Cruz, CA, USA) and then for 1 h at room temperature with Envision rabbit HRP (1:1, K4003, Dako, Carpinteria, CA, USA). Diaminobenzidine (Dako, Carpinteria, CA, USA) was used as a chromogen and hematoxylin as a counterstain.

AMH immunostaining protocol

Sections were deparaffinized with Histosafe (Yvsolab SA, Turnhout, Belgium) and rehydrated in 2-propanol (Merck, Darmstadt, Germany). After blocking endogenous peroxidase activity with 3% H_2O_2 and demasking in citrate buffer (pH 6) for 75 min at 98 °C, nonspecific binding sites were blocked by incubation with normal goat serum for 30 min. Sections were first incubated overnight at 4 °C with mouse anti-human AMH (1:100, MCA2246, Serotec, Gentaur, Kampenhout, Belgium) and then for 1 h at room temperature with Envision mouse HRP (1:2, K4001, Dako, Carpinteria, CA, USA). Diaminobenzidine (Dako, Carpinteria, CA, USA) was used as a chromogen and hematoxylin as a counterstain.

AMHRII immunostaining protocol

Sections were deparaffinized with Histosafe (Yvsolab SA, Turnhout, Belgium) and rehydrated in 2-propanol (Merck, Darmstadt, Germany). After blocking endogenous peroxidase activity with 3% H_2O_2 and demasking in citrate buffer (pH 6) for 75 min at 98 °C, nonspecific binding sites were blocked by incubation with normal goat serum for 30 min. Sections were first incubated overnight at 4 °C with rabbit polyclonal AMHRII (1:200, 36093 Signalway Antibody, College Park, MD, USA) and then for 1 h at room temperature with Envision rabbit HRP (1:2, K4003, Dako, Carpinteria, CA, USA). Diaminobenzidine (Dako, Carpinteria, CA, USA) was used as a chromogen and hematoxylin as a counterstain.

Supplementary Table S1. Total and per patient follicle count in non-grafted controls, the 2-step/ASCs+OT group, and the OT group.

Patients.	Non-grafted controls				6-month transplantation					
	Total	Primordial	Primary	Secondary		Total	Primordial	Primary	Secondary	Antral
1	52	46	6	0	2-step/ASCs+OT	3	2	1	0	0
					OT	0	0	0	0	0
2	10	8	2	0	2-step/ASCs+OT	43	27	3	5	8
					OT	0	0	0	0	0
3	54	29	13	12	2-step/ASCs+OT	15	5	4	5	1
					OT	18	1	6	7	2
4	64	51	12	1	2-step/ASCs+OT	60	30	12	9	9
					OT	19	6	6	5	2
5	14	7	5	2	2-step/ASCs+OT	0	0	0	0	0
					OT	1	0	0	1	0
6	12	10	2	0	2-step/ASCs+OT	47	32	4	7	4
					OT	19	5	0	7	7
7	4	4	0	0	2-step/ASCs+OT	1	1	0	0	0
					OT	2	1	0	1	0
	210	155	40	15		169	97	24	26	22
						57	13	12	21	11
